# Optimal Designs of the Median Run Length Based Double Sampling *X̄* Chart for Minimizing the Average Sample Size

**DOI:** 10.1371/journal.pone.0068580

**Published:** 2013-07-25

**Authors:** Wei Lin Teoh, Michael B. C. Khoo, Sin Yin Teh

**Affiliations:** 1 Department of Physical and Mathematical Science, Faculty of Science, Universiti Tunku Abdul Rahman, Jalan Universiti, Bandar Barat, Kampar, Perak, Malaysia; 2 School of Mathematical Sciences, Universiti Sains Malaysia, Penang, Malaysia; 3 School of Management, Universiti Sains Malaysia, Penang, Malaysia; National Institute of Environmental and Health Sciences, United States of America

## Abstract

Designs of the double sampling (DS) 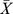 chart are traditionally based on the average run length (ARL) criterion. However, the shape of the run length distribution changes with the process mean shifts, ranging from highly skewed when the process is in-control to almost symmetric when the mean shift is large. Therefore, we show that the ARL is a complicated performance measure and that the median run length (MRL) is a more meaningful measure to depend on. This is because the MRL provides an intuitive and a fair representation of the central tendency, especially for the rightly skewed run length distribution. Since the DS 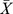 chart can effectively reduce the sample size without reducing the statistical efficiency, this paper proposes two optimal designs of the MRL-based DS 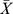 chart, for minimizing (i) the in-control average sample size (ASS) and (ii) both the in-control and out-of-control ASSs. Comparisons with the optimal MRL-based EWMA 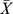 and Shewhart 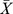 charts demonstrate the superiority of the proposed optimal MRL-based DS 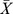 chart, as the latter requires a smaller sample size on the average while maintaining the same detection speed as the two former charts. An example involving the added potassium sorbate in a yoghurt manufacturing process is used to illustrate the effectiveness of the proposed MRL-based DS 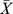 chart in reducing the sample size needed.

## Introduction

Statistical process control (SPC) is a powerful collection of statistical tools for achieving process stability. SPC is based on sound underlying principles, which is easy to use and can be applied in the manufacturing and service processes, such as in the food industries, automobile industries, as well as health-care and public-health surveillance [1]. A control chart is one of the valuable quality improvement techniques in SPC that can be used to attain process stability and reduce process variability over time. Since the double sampling (DS) 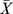 chart was introduced by Croasdale [2] in 1974, the DS scheme has been studied extensively among researchers. By applying the concept of double sampling plans, Daudin [3] suggested an improved DS 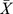 chart which incorporates both the ideas of variable sampling interval (VSI) and variable sample size (VSS). Unlike the VSI procedure, two successive samples are taken in the DS procedure without any intervening time; where, both the first and second samples of the DS chart are taken from the same population.

Recently, considerable efforts have been undertaken on the research of various DS type charts, which can be categorized into the DS 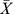 type, DS *S* type and other DS type control charts. Costa and Machado [4], Khoo et al. [5] and Torng et al. [6] investigated the DS 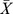 type charts for monitoring the process mean. Works on the DS *S* type charts for monitoring the process variance were discussed by He and Grigoryan [7], [8] and Lee et al. [9], [10]. Other DS type charts are the joint DS 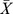 and *S* chart, proposed by He and Grigoryan [11], for a simultaneous monitoring of the process mean and variance, as well as the DS *np* chart for attributes, suggested by Rodrigues et al. [12].

It is known that the DS 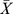 chart not only maintains the simplicity of the Shewhart 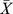 chart, but the former also improves the statistical efficiency of the latter in detecting process mean shifts, besides reducing the sample size [13]. Compared to the Shewhart 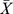 chart, He et al. [14] claimed that the sample size of the DS 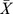 chart dramatically decreases to nearly 50% when the process is in-control. In addition, the DS 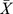 chart has an advantage of having a lower total sample size when the incoming quality is either very excellent or very poor [15]. This is because only the first sample is required to sentence the process as either in-control or out-of-control. Therefore, the DS scheme is an appropriate choice for process monitoring with destructive testing and high inspection costs [16]. In view of these advantages, many researchers (see [2], [3], [7], [8], [14]) focused on proposing the DS chart for minimizing the in-control average sample size (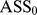). Hsu [17], [18] claimed that the conclusion made by He et al. [14] and He and Grigoryan [7] is questionable since the out-of-control average sample size (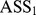) is disregarded when comparing the various charts' performances. Accordingly, Lee et al. [10] modified the design model of He and Grigoryan [8] to propose the DS *S* chart which minimizes both the 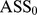 and 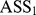.

The average run length (ARL) has been traditionally used as a sole measure of a control chart's performance. The sole reliance on the ARL has been widely criticized by Das [19], Gan [20] and Golosnoy and Schmid [21]. This criticism comes from two concerns [1]. First, the value of the standard deviation of the run length (SDRL) is quite large. Second, the run length distribution is highly skewed. Furthermore, Thaga [22] stated that only a fraction of a chart's behavior is reflected by the size of the ARL. Therefore, misleading conclusion is drawn based on the ARL as it is not necessarily a typical run length. On the other hand, the median run length (MRL) is a more credible measure of a chart's performance since it is less affected by the skewness of the run length distribution [20], [23]. The MRL is the 50^th^ percentile of the run length distribution, representing “half of the time” [24]. For example, when the in-control MRL (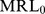) is 250, a practitioner can claim that a false alarm will occur by the 250^th^ sample in half of the time; while an out-of-control MRL (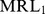) of 10 means that for this particular shift, there is a 50% chance that an out-of-control signal will be produced in not later than the 10^th^ sample. For ease of interpretation and a better understanding of a chart's performance, Gan [20], Golosnoy and Schmid [21], Maravelakis et al. [23], Khoo et al. [25] and Low et al. [26] have all advocated using MRL as an alternative measure to evaluate a chart's performance.

Similar to other charts, the ARL is widely used in the literature as a performance and design criteria of the DS 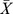 chart. However, when the run length distribution is highly skewed to the right, especially for an in-control process or when the shift is small, we show that the ARL is a peculiar measure of a typical chart's performance and that the MRL is a more meaningful quantity to rely on. Keeping this in mind, two new optimal design procedures for the MRL-based DS 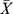 chart by minimizing the (i) 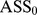 and (ii) 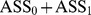 are developed in this paper. In this paper, the average sample size (ASS) is chosen as the objective function of the optimal design models. This is because these optimal design models are applicable to small enterprises which have a low production volume or are useful for monitoring destructive testing processes. The reason for minimizing the 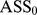 is due to the fact that the process will operate in the in-control state for most of the time [1], [3], [8], [14]. Hsu [17], [18] stated that the ASS for both the in-control and out-of-control situations should be taken into consideration when designing a control chart. Therefore, the second optimal design, i.e. minimizing the 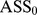 and 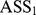, is proposed in accordance with the argument of Hsu [17], [18]. Consequently, a smaller sample size is used and this leads to a substantial reduction of inspection and sampling costs.

The rest of this paper is organized as follows: The DS 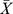 chart's procedure and its run length properties are briefly introduced in Section 2. Section 3 examines the performance of the DS 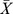 chart, in terms of the percentiles of the run length distribution, ARL and ASS. Two optimal designs of the MRL-based DS 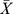 chart, for minimizing the (i) 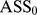 and (ii) 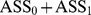 are proposed in Section 4. Besides providing the optimal chart parameters for the MRL-based DS 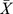 chart, Section 5 compares the sample-size performance of the optimal MRL-based DS 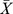, EWMA 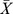 and Shewhart 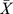 charts. An illustrative example on the construction of the optimal MRL-based DS 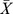 chart is given in Section 6. Conclusions are drawn in Section 7.

## The DS 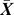 Control Chart for Monitoring the Process Mean

**Figure 1:**
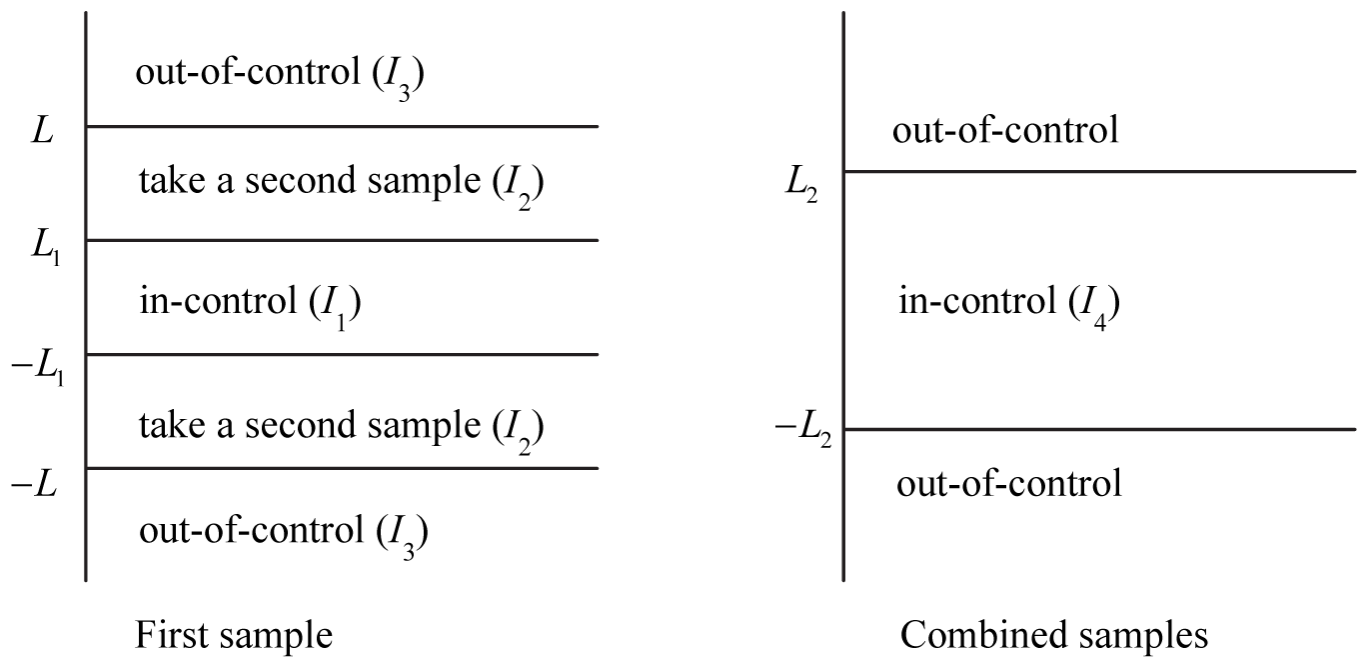
Schematic representation of the DS 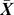 chart's operation. The DS 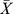 chart consists of two stages, i.e. the first-sample stage and the combined-sample stage.

Assume that the observations of the quality characteristic *X* are independent and follow an identical normal 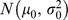 distribution with the in-control mean 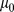 and variance 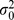. We further assume that 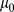 and 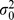 are known. By referring to Figure 1, let 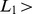
0 and 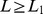 be the warning and control limits of the first-sample stage, respectively; while 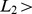
0 is the control limit of the combined-sample stage. The regions of the DS 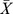 chart can be divided into 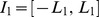, 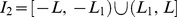, 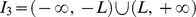 and 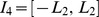. The charting procedure of the DS 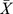 chart is as follows:

1. Determine the limits 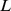, 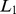 and 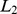.
2. Take a first sample of size 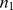 and calculate the first sample mean 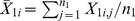. Here, 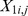 for 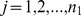, is the *j* ^th^ observation at the *i* ^th^ sampling time of the first sample.
3. Declare the process as in-control if 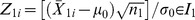. Then the control flow returns to Step (2).
4. Declare the process as out-of-control if 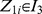 and then proceed to Step (8).
5. Take a second sample of size 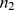 from the same population as the first sample if 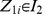. Then compute the second sample mean 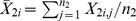. Here, 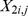 for 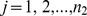, is the *j* ^th^ observation at the *i* ^th^ sampling time of the second sample.
6. Calculate the combined-sample mean 
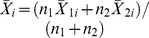
 at the *i* ^th^ sampling time.
7. Declare the process as in-control if 
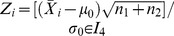
; otherwise, declare the process as out-of-control and advance to Step (8).
8. Issue an out-of-control signal at the *i* ^th^ sampling time to indicate a process mean shift.
9. Investigate and remove assignable cause(s) and then return to Step (2).

Note that the *i* ^th^ sampling time refers to the *i* ^th^ time when either only the first sample of size 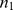 or both the first and second samples of size 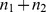, are collected.

Let 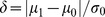 be the size of a standardized mean shift, where 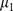 is the out-of-control mean. If 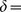
0, the process is considered as in-control; otherwise, it is deemed as out-of-control. Let 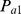 and 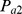 represent the probabilities that the process remains in-control “by the first sample” and “after taking the second sample”, respectively. Then, 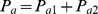 is the probability that the process is regarded as in-control, where 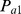 and 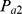 are given as [3]

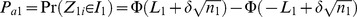
(1)and
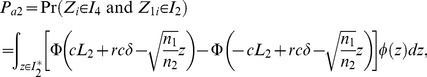
(2)respectively, where 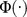 and 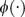 are the standard normal cumulative distribution function (cdf) and standard normal probability density function (pdf), respectively. In Equation (2), 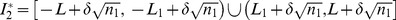, 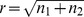 and 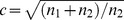.

Let RL represents the run length which is the number of samples collected until the first out-of-control signal is detected. The RL distribution of a Shewhart 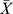 chart follows a geometric distribution when the chart's control limits are known constants and the plotted statistics are independently and identically distributed random variables [1]. Since the DS 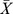 chart is a two-stage Shewhart 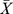 chart, all the RL properties of the DS 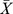 chart can be characterized by those of the geometric distribution. Hence, the cdf 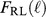 of the RL for the DS 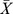 chart, defined for 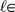
{1, 2, 3, …}, is calculated as
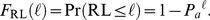
(3)It follows that the MRL of the DS 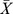 chart is equal to [20]

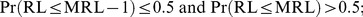
(4)while the other 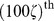 percentiles of the RL distribution are computed as the value 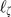, such that
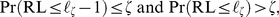
(5)where 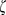 is in the range of 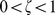.

Daudin [3] also showed that the ARL of the DS 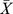 chart is
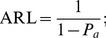
(6)while the ASS at each sampling time is defined as
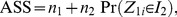
(7)where 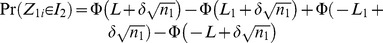.

## Performance of the DS 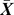 Chart Based on the Percentiles of the Run Length Distribution, ARL and ASS

Palm [24] claimed that a practitioner is more interested in the percentiles of the RL distribution as they provide additional and detailed information regarding the expected behavior of the RL. Therefore, we investigate the performance of the optimal ARL-based DS 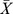 chart for minimizing 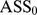, in terms of ARL, ASS and the percentiles of the RL distribution. Table 1 summarizes these performance measures for the DS 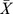 chart when the in-control ARL, 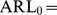
370.0 and the out-of-control ARL, 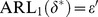. Here, 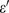 is the desired 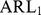 value corresponding to a shift 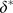. The optimization procedure given by Daudin [3] is applied here. The 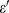 value is specified as the 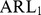 value of the optimal EWMA 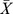 chart, where the 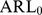 value and the sample size (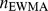) of this EWMA chart are set as 370.0 and (3, 5), respectively. In Table 1, the optimal (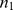, 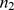, 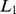, 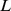, 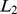) combinations of the ARL-based DS 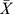 chart for minimizing 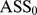 are obtained such that 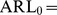
370.0 and 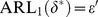. Here, the 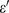 values in Table 1 are selected so that when 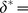
0.5, 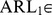
{11.9, 8.1} for 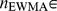
{3, 5}; and when 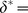
1.0, 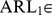
{4.2, 2.8} for 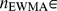
{3, 5}. These optimal chart parameters are used to calculate the ARL, ASS and the percentiles of the RL distribution based on the formulae shown in Section 2. Note that the 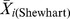 in Table 1 represents the sample size of the ARL-based Shewhart 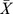 chart, matching approximately a similar design of the ARL-based DS 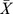 chart.

**Table 1:** ARLs, ASSs and percentiles of the run length distribution for the DS 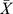 chart when 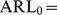
370.0 and 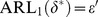.

						Percentiles of the run length distribution										
*δ* ^*^	*n*_EWMA_	*n_X_*_-bar_	*δ*	ARL	ASS	5^th^	10^th^	20^th^	30^th^	40^th^	50^th^	60^th^	70^th^	80^th^	90^th^	95^th^
0.5	3	11	*n*_1_ = 2, *n*_2_ = 18, *L*_1_ = 1.847, *L* = 5.885, *L*_2_ = 2.368													
			0.00	370.0	3.165	19	39	83	132	189	257	339	445	595	851	1107
			0.25	57.8	3.467	3	7	13	21	30	40	53	70	93	133	172
			0.50	11.9	4.384	1	2	3	5	6	8	11	14	19	27	35
			1.00	3.0	7.995	1	1	1	1	2	2	3	3	5	6	8
			1.50	1.6	12.943	1	1	1	1	1	1	1	2	2	3	4
			2.00	1.2	17.041	1	1	1	1	1	1	1	1	1	2	2
			3.00	1.0	18.946	1	1	1	1	1	1	1	1	1	1	1
	5	14	*n*_1_ = 3, *n*_2_ = 17, *L*_1_ = 1.647, *L* = 5.796, *L*_2_ = 2.599													
			0.00	370.0	4.691	19	39	83	132	189	257	339	445	595	851	1107
			0.25	46.7	5.228	3	5	11	17	24	33	43	56	75	107	139
			0.50	8.1	6.796	1	1	2	3	4	6	7	10	13	18	23
			1.00	1.9	12.080	1	1	1	1	1	1	2	2	3	4	4
			1.50	1.2	17.084	1	1	1	1	1	1	1	1	1	2	2
			2.00	1.0	19.244	1	1	1	1	1	1	1	1	1	1	1
			3.00	1.0	15.334	1	1	1	1	1	1	1	1	1	1	1
1.0	3	5	*n*_1_ = 1, *n*_2_ = 8, *L*_1_ = 1.525, *L* = 5.977, *L*_2_ = 2.591													
			0.00	370.0	2.018	19	39	83	132	189	257	339	445	595	851	1107
			0.25	103.2	2.112	6	11	23	37	53	72	95	124	166	237	308
			0.50	23.7	2.392	2	3	6	9	12	17	22	28	38	54	70
			1.00	4.2	3.444	1	1	1	2	2	3	4	5	6	9	11
			1.50	2.1	4.929	1	1	1	1	1	2	2	2	3	4	5
			2.00	1.5	6.462	1	1	1	1	1	1	1	2	2	3	3
			3.00	1.1	8.427	1	1	1	1	1	1	1	1	1	1	2
	5	7	*n*_1_ = 2, *n*_2_ = 9, *L*_1_ = 1.659, *L* = 5.899, *L*_2_ = 2.646													
			0.00	370.0	2.875	19	39	83	132	189	257	339	445	595	851	1107
			0.25	82.7	3.063	5	9	19	30	42	57	76	99	133	190	247
			0.50	16.7	3.617	1	2	4	6	9	12	15	20	27	38	49
			1.00	2.8	5.641	1	1	1	1	2	2	3	3	4	6	7
			1.50	1.5	8.104	1	1	1	1	1	1	1	2	2	3	3
			2.00	1.1	9.901	1	1	1	1	1	1	1	1	1	2	2
			3.00	1.0	10.517	1	1	1	1	1	1	1	1	1	1	1

From Table 1, we observe that the difference between the values of ARL and MRL is large when 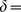
0 and it diminishes as 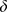 increases. This indicates that the shape and the skewness of the RL distribution change with the magnitude of the process mean shift 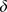. Also, the ARLs shown in Table 1 are all larger than the MRLs (i.e. 50^th^ percentile of the RL distribution) when 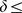
2.0. This is due to the fact that in a right-skewed RL distribution, the value of the average of the RL is greater than the median of the RL. Thus, the MRL is a better representation of the central tendency compared to the ARL. Note that the ARL only measures the expected run length and does not indicate the likelihood of getting a signal by a certain probability. For example, when 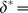
1.0, 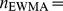
3 and 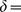
0.25 are considered, there could exist a risk where a practitioner falsely interprets that an out-of-control is detected by the 103^rd^ sampling time (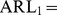
103.2) in 50% of the time, but in actual fact, this event occurs noticeably earlier, i.e. by the 72^nd^ sampling time (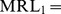
72).

An advantage of computing the lower percentiles (e.g. 5^th^, 10^th^ and 20^th^ percentiles) of the RL distribution for 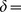
0 is that it allows the probability analysis of early false signals to be carried out. From Table 1, we notice that even when the value of 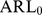 is large, the lower percentiles are remarkably shorter. This suggests that even when the false alarm rate (FAR = 0.0027) is low, a relatively large percent of false signals occur very early in the process monitoring. The computation of the higher percentiles (e.g. 80^th^, 90^th^ and 95^th^ percentiles) of the RL distribution also provides some useful information to a practitioner. For instance, when 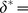
0.5, 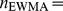
5 and 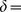
1.0 are considered, a practitioner can state with a 90% confidence that a shift with magnitude 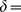
1.0 is signaled by the fourth sampling time.

Table 1 provides clear evidence that the in-control RL distribution is highly skewed and that the skewness of the RL distribution changes with 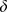. Therefore, interpretation based on the average of the RL (or ARL) with respect to a highly skewed RL distribution is certainly misleading compared to the case if the RL distribution is symmetric. When the associated RL distribution has different levels of skewness as 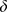 changes, the MRL provides a more meaningful performance measure for the DS 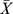 chart. Along this line, we are motivated to propose two optimal designs (see Section 4) of the MRL-based DS 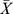 chart.

## Optimal Designs of the MRL-Based DS 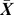 Chart

The optimal designs of the MRL-based DS 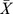 chart having the smallest (i) 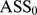 and (ii) both the 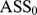 and 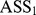, are proposed in Sections 4.1 and 4.2, respectively. The optimization programs are written using the ScicosLab software (www.scicoslab.org). It is not easy to optimally determine the five charting parameters, i.e. 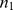, 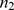, 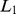, 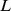 and 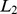 of the DS 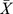 chart. Therefore, these optimal chart parameters are searched through the implementation of the Nelder Mead's nonlinear optimization algorithm [27]. Since the sample sizes 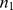 and 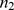 are parameters to be optimized, we need to limit the allowable upper bound, i.e. 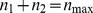. Thus, 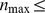
20 is fixed in this paper because it is a common practice in industries to use small and moderate sample sizes.

### 4.1 Minimizing the in-control ASS

The proposed optimal design of the MRL-based DS 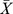 chart for minimizing the 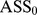 is illustrated as follows:
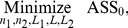
(8)subject to

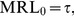
(9)where 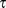 is the desired in-control MRL.

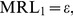
(10)where 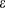 is the desired out-of-control MRL corresponding to a shift 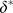.

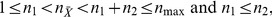
(11)where 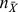 is the sample size of the MRL-based Shewhart 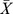 chat, matching approximately a similar design of the MRL-based DS 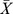 chart.

By applying the optimization model (8)–(11), the steps for obtaining the optimal MRL-based DS 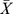 chart's parameters (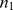, 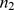, 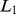, 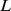, 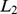) are demonstrated as follows:

1. Specify the desired values of 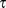, 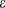, 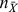, 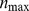 and 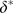.
2. Search the parameters 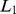, 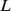 and 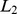 for all the (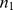, 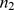) pairs selected based on constraint (11). A nonlinear equation solver is used to determine these three parameters. Note that for any given value of 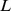, the values of 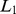 and 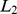 are adjusted simultaneously to satisfy both the constraints (9) (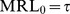) and (10) (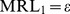). At the end of this step, all the possible (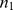, 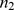, 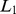, 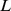, 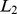) combinations fulfilling constraints (9)–(11) are obtained.
3. Identify the optimal (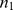, 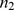, 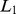, 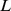, 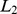) combination which has the smallest value of 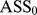 from all the chart-parameter combinations found in Step (2).

For example, when 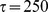, 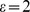, 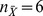, 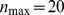 and 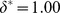, the output listing and the optimal (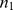, 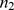, 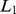, 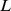, 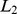) combination (see the last row of the output listing) are obtained as

n1 n2 L1    L   L2  MRL0 MRL1 ASS0 ASS1

1 6 0.869930 5.027832 2.857255 250 2 3.306028 4.494778

1 7 1.142700 5.592773 2.763021 250 2 2.772141 4.215309

1 8 1.283047 5.327637 2.692279 250 2 2.595803 4.198212

.

.

.

5 13 2.774352 3.005859 2.985578 250 2 5.037477 5.968226

5 14 2.779625 3.035400 2.489431 250 2 5.042560 6.138543

5 15 2.780030 3.035889 2.454888 250 2 5.045557 6.219900

2 7 1.787 5.133  2.633  250 2 2.517 4.486

The output listing is not shown completely here as there are 66 (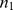, 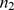) pairs with the corresponding smallest 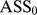 value (see the 8^th^ column of each row in the output listing), for each (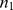, 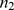) pair.

### 4.2 Minimizing both the in-control and out-of-control ASSs

Hsu [17], [18] indicated that the optimal design of a control chart should take into consideration both the in-control and out-of-control situations. Therefore, in order to provide the best performance of the MRL-based DS 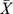 chart for a specified mean shift 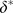, two objective functions, i.e. minimizing 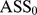 and 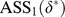 are proposed in this section. The weighting average method suggested by Zadeh [28] is used to integrate these two objective functions. This weighting average method allows us to assign a weight to each objective function and then combine them into a single objective function. Since the performance of both the in-control and out-of-control cases are equally important, we let the weights of the 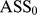 and 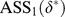 equal to each other. Hence, the integrated objective function of this proposed optimal design model is simplified to the minimization of 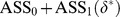.

The proposed optimal design of the MRL-based DS 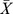 chart to minimize both the 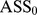 and 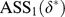, which is modeled as a nonlinear minimization problem, is mathematically expressed as follows:
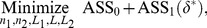
(12)subject to

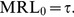
(13)

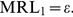
(14)

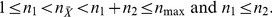
(15)The design procedure of the optimization model (12)–(15) is similar to that presented in Step (1) to Step (3) of Section 4.1. The only difference is that we are minimizing 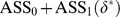 instead of 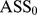.

## Comparative Studies

The performance of the optimal MRL-based DS 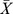 chart is now compared with the Shewhart 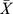 and optimal EWMA 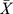 charts. The 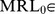
{250, 500} and various values of 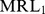 corresponding to 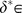
{0.50, 0.75, 1.00, 1.25, 1.50, 1.75, 2.00, 2.50, 3.00} are considered. Thus, the three charts are compared based on their sample-size performance. Note that only moderate and large 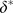 are considered in this paper because in many real industrial applications, small shifts in the process are usually not desirable to be detected in order to avoid too frequent process interruptions [5], [29].

For the Shewhart 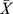 chart, the upper control limit 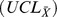, lower control limit 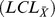 and center line 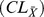 are computed as [1]

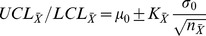
(16a)and
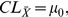
(16b)respectively, where 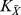 is a multiplier controlling the width of both the 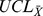 and 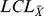.

For the EWMA 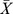 chart, the plotting statistics 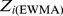 is expressed as [1]

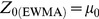
(17a)and
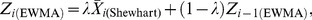
(17b)where 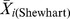 is the sample mean at the *i* ^th^ sampling time and 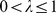. Then the upper and lower control limits, i.e. 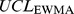 and 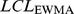, respectively, as well as the center line 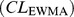 are defined as follows [1]:
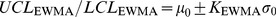
(18a)and
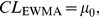
(18b)respectively, where 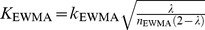 with the multiplier 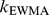 to be ascertained.

In this study, 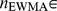
{3, 5, 7} are considered. The optimization procedure shown in Khoo et al. [25] is used to optimally design the MRL-based EWMA 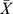 chart for minimizing the 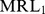.

### 5.1 Study 1: The DS 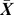 chart for minimizing the 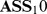


In Study 1, we compare the sample size performance of the optimal MRL-based EWMA 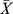, Shewhart 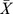 and DS 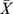 charts. Tables 2 and 3 present the optimal chart parameters for these three charts, together with their corresponding values of 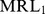 and sample size (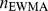, 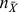 or ASS). For the EWMA 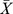 chart, the optimal parameters (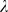, 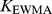) and the corresponding (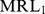, 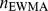) values are shown in the first and second rows of each cell, respectively. Meanwhile, the charting constant 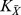 and the corresponding (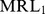, 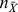) values of the Shewhart 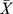 chart are listed in the first and second rows of each cell, respectively. For the DS 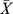 chart, the optimal combination (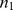, 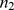, 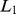, 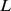, 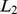) is presented in the first and second rows of each cell, while the corresponding (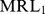, 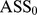, 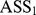) values are presented in the third row of each cell.

**Table 2:** Optimal chart parameters for the EWMA 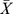, Shewhart 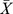 and DS 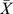 charts, together with their corresponding values of 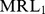 and sample size when 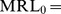
250 and 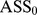 is minimized.

	*n*_EWMA_ = 3			*n*_EWMA_ = 5			*n*_EWMA_ = 7		
	EWMA *X*-bar	Shewhart *X*-bar	DS *X*-bar	EWMA *X*-bar	Shewhart *X*-bar	DS *X*-bar	EWMA *X*-bar	Shewhart *X*-bar	DS *X*-bar
	(*λ*, *K*_EWMA_)	*K_X_*_-bar_	(*n*_1_, *n*_2_,	(*λ*, *K*_EWMA_)	*K_X_*_-bar_	(*n*_1_, *n*_2_,	(*λ*, *K*_EWMA_)	*K_X_*_-bar_	(*n*_1_, *n*_2_,
	(MRL_1_, *n*_EWMA_)	(MRL_1_, *n_X_*_-bar_)	*L*_1_, *L*, *L*_2_)	(MRL_1_, *n*_EWMA_)	(MRL_1_, *n_X_*_-bar_)	*L*_1_, *L*, *L*_2_)	(MRL_1_, *n*_EWMA_)	(MRL_1_, *n_X_*_-bar_)	*L*_1_, *L*, *L*_2_)
***δ*** ^*^			(MRL_1_, ASS_0_, ASS_1_)			(MRL_1_, ASS_0_, ASS_1_)			(MRL_1_, ASS_0_, ASS_1_)
0.50	(0.175, 0.505)	2.992	(1, 19,	(0.300, 0.548)	2.992	(2, 18,	(0.300, 0.463)	2.992	(3, 17,
	(10, 3)	(10, 9)	1.787, 5.561, 2.261)	(7, 5)	(7, 12)	1.742, 5.171, 2.431)	(5, 7)	(5, 14)	1.594, 4.955, 2.617)
			(10, 2.405, 3.092)			(7, 3.467, 4.835)			(5, 4.885, 7.083)
0.75	(0.300, 0.707)	2.992	(1, 10,	(0.550, 0.820)	2.992	(1, 13,	(0.550, 0.693)	2.992	(2, 13,
	(6, 3)	(6, 6)	1.794, 5.939, 2.373)	(4, 5)	(4, 8)	1.583, 5.163, 2.463)	(3, 7)	(3, 9)	1.728, 5.248, 2.513)
			(6, 1.728, 2.537)			(4, 2.475, 3.760)			(3, 3.091, 5.312)
1.00	(0.550, 1.058)	2.992	(1, 6,	(0.550, 0.820)	2.992	(2, 7,	(0.550, 0.693)	2.992	(2, 7,
	(4, 3)	(4, 4)	1.818, 5.337, 2.477)	(2, 5)	(2, 6)	1.787, 5.133, 2.633)	(2, 7)	(2, 6)	1.787, 5.133, 2.633)
			(4, 1.415, 2.255)			(2, 2.517, 4.486)			(2, 2.517, 4.486)
1.25	(0.550, 1.058)	2.992	(1, 4,	(0.550, 0.820)	2.992	(1, 5,	(0.800, 0.923)	2.992	(2, 6,
	(3, 3)	(4, 3)	1.919, 5.145, 2.521)	(2, 5)	(2, 4)	1.615, 4.700, 2.634)	(1, 7)	(1, 6)	1.618, 4.992, 2.744)
			(3, 1.220, 2.010)			(2, 1.531, 2.796)			(1, 2.634, 5.356)
1.50	(0.550, 1.058)	2.992	(1, 3,	(0.550, 0.820)	2.992	(1, 5,	(0.550, 0.693)	2.992	(1, 5,
	(2, 3)	(2, 3)	1.893, 5.047, 2.618)	(1, 5)	(1, 4)	1.366, 5.110, 2.738)	(1, 7)	(1, 4)	1.366, 5.110, 2.738)
			(2, 1.175, 2.042)			(1, 1.859, 3.775)			(1, 1.859, 3.775)
1.75	(0.550, 1.058)	2.992	(1, 2,	(0.550, 0.820)	2.992	(1, 3,	(0.550, 0.693)	2.992	(1, 3,
	(2, 3)	(2, 2)	2.206, 4.603, 2.545)	(1, 5)	(1, 3)	1.581, 5.164, 2.758)	(1, 7)	(1, 3)	1.581, 5.164, 2.758)
			(2, 1.055, 1.644)			(1, 1.342, 2.702)			(1, 1.342, 2.702)
2.00	(0.550, 1.058)	2.992	(1, 3,	(0.550, 0.820)	2.992	(1, 3,	(0.550, 0.693)	2.992	(1, 3,
	(1, 3)	(1, 3)	1.970, 5.431, 2.573)	(1, 5)	(1, 3)	1.970, 5.431, 2.573)	(1, 7)	(1, 3)	1.970, 5.431, 2.573)
			(1, 1.146, 2.535)			(1, 1.146, 2.535)			(1, 1.146, 2.535)
2.50	(0.550, 1.058)	2.992	(1, 2,	(0.550, 0.820)	2.992	(1, 2,	(0.550, 0.693)	2.992	(1, 2,
	(1, 3)	(1, 2)	2.485, 3.061, 2.893)	(1, 5)	(1, 2)	2.485, 3.061, 2.893)	(1, 7)	(1, 2)	2.485, 3.061, 2.893)
			(1, 1.021, 1.437)			(1, 1.021, 1.437)			(1, 1.021, 1.437)
3.00	(0.550, 1.058)	2.992	(1, 2,	(0.550, 0.820)	2.992	(1, 2,	(0.550, 0.693)	2.992	(1, 2,
	(1, 3)	(1, 2)	2.992, 3.437, 0.000)	(1, 5)	(1, 2)	2.992, 3.437, 0.000)	(1, 7)	(1, 2)	2.992, 3.437, 0.000)
			(1, 1.004, 1.344)			(1, 1.004, 1.344)			(1, 1.004, 1.344)

**Table 3:** Optimal chart parameters for the EWMA 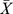, Shewhart 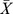 and DS 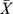 charts, together with their corresponding values of 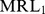 and sample size when 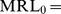
500 and 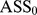 is minimized.

	*n*_EWMA_ = 3			*n*_EWMA_ = 5			*n*_EWMA_ = 7		
	EWMA *X*-bar	Shewhart *X*-bar	DS *X*-bar	EWMA *X*-bar	Shewhart *X*-bar	DS *X*-bar	EWMA *X*-bar	Shewhart *X*-bar	DS *X*-bar
	(*λ*, *K*_EWMA_)	*K_X_*_-bar_	(*n*_1_, *n*_2_,	(*λ*, *K*_EWMA_)	*K_X_*_-bar_	(*n*_1_, *n*_2_,	(*λ*, *K*_EWMA_)	*K_X_*_-bar_	(*n*_1_, *n*_2_,
	(MRL_1_, *n*_EWMA_)	(MRL_1_, *n_X_*_-bar_)	*L*_1_, *L*, *L*_2_)	(MRL_1_, *n*_EWMA_)	(MRL_1_, *n_X_*_-bar_)	*L*_1_, *L*, *L*_2_)	(MRL_1_, *n*_EWMA_)	(MRL_1_, *n_X_*_-bar_)	*L*_1_, *L*, *L*_2_)
***δ*** ^*^			(MRL_1_, ASS_0_, ASS_1_)			(MRL_1_, ASS_0_, ASS_1_)			(MRL_1_, ASS_0_, ASS_1_)
0.50	(0.175, 0.548)	3.198	(1, 19,	(0.175, 0.424)	3.198	(2, 18,	(0.300, 0.499)	3.198	(3, 17,
	(12, 3)	(13, 10)	1.767, 5.024, 2.558)	(8, 5)	(8, 14)	1.650, 5.147, 2.760)	(6, 7)	(6, 16)	1.506, 5.586, 2.915)
			(12, 2.466, 3.170)			(8, 3.781, 5.278)			(6, 5.246, 7.591)
0.75	(0.238, 0.659)	3.198	(1, 14,	(0.300, 0.590)	3.198	(2, 14,	(0.550, 0.721)	3.198	(2, 16,
	(6, 3)	(6, 7)	1.842, 5.116, 2.560)	(4, 5)	(4, 9)	1.916, 5.084, 2.664)	(3, 7)	(3, 11)	1.729, 5.945, 2.743)
			(6, 1.917, 2.991)			(4, 2.775, 4.765)			(3, 3.342, 6.075)
1.00	(0.550, 1.133)	3.198	(1, 8,	(0.550, 0.878)	3.198	(1, 9,	(0.550, 0.721)	3.198	(2, 9,
	(4, 3)	(4, 5)	1.844, 5.038, 2.677)	(3, 5)	(3, 6)	1.651, 5.409, 2.760)	(2, 7)	(2, 8)	1.805, 5.292, 2.836)
			(4, 1.521, 2.612)			(3, 1.889, 3.354)			(2, 2.640, 5.137)
1.25	(0.550, 1.133)	3.198	(1, 5,	(0.550, 0.878)	3.198	(1, 7,	(0.800, 0.959)	3.198	(2, 8,
	(3, 3)	(3, 4)	1.910, 5.297, 2.755)	(2, 5)	(2, 5)	1.686, 5.758, 2.794)	(1, 7)	(1, 7)	1.670, 5.274, 2.921)
			(3, 1.281, 2.277)			(2, 1.643, 3.333)			(1, 2.760, 6.313)
1.50	(0.550, 1.133)	3.198	(1, 4,	(0.800, 1.167)	3.198	(1, 6,	(0.550, 0.721)	3.198	(1, 6,
	(2, 3)	(2, 4)	1.918, 5.294, 2.809)	(1, 5)	(1, 5)	1.381, 5.582, 2.949)	(1, 7)	(1, 5)	1.381, 5.582, 2.949)
			(2, 1.220, 2.352)			(1, 2.003, 4.295)			(1, 2.003, 4.295)
1.75	(0.550, 1.133)	3.198	(1, 3,	(0.550, 0.878)	3.198	(1, 4,	(0.550, 0.721)	3.198	(1, 4,
	(2, 3)	(2, 3)	2.236, 5.253, 2.704)	(1, 5)	(1, 4)	1.639, 4.736, 2.934)	(1, 7)	(1, 4)	1.639, 4.736, 2.934)
			(2, 1.076, 1.940)			(1, 1.405, 3.172)			(1, 1.405, 3.172)
2.00	(0.800, 1.507)	3.198	(1, 3,	(0.550, 0.878)	3.198	(1, 3,	(0.550, 0.721)	3.198	(1, 3,
	(1, 3)	(1, 3)	1.934, 5.785, 2.877)	(1, 5)	(1, 3)	1.934, 5.785, 2.877)	(1, 7)	(1, 3)	1.934, 5.785, 2.877)
			(1, 1.159, 2.579)			(1, 1.159, 2.579)			(1, 1.159, 2.579)
2.50	(0.550, 1.133)	3.198	(1, 2,	(0.550, 0.878)	3.198	(1, 2,	(0.550, 0.721)	3.198	(1, 2,
	(1, 3)	(1, 2)	2.482, 3.399, 2.899)	(1, 5)	(1, 2)	2.482, 3.399, 2.899)	(1, 7)	(1, 2)	2.482, 3.399, 2.899)
			(1, 1.025, 1.646)			(1, 1.025, 1.646)			(1, 1.025, 1.646)
3.00	(0.550, 1.133)	3.198	(1, 2,	(0.550, 0.878)	3.198	(1, 2,	(0.550, 0.721)	3.198	(1, 2,
	(1, 3)	(1, 2)	3.000, 3.259, 2.578)	(1, 5)	(1, 2)	3.000, 3.259, 2.578)	(1, 7)	(1, 2)	3.000, 3.259, 2.578)
			(1, 1.003, 1.205)			(1, 1.003, 1.205)			(1, 1.003, 1.205)

In this study, all the three charts are designed to have a similar sensitivity for a particular 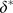, i.e. by having a similar 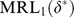 value as that of the optimal MRL-based EWMA 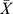 chart when 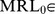
{250, 500}. In particular, the optimal combination (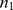, 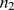, 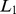, 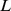, 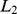) of the DS 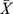 chart is obtained such that 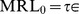
{250, 500} (constraint (9)) and 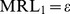 (constraint (10)) for a 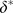. Here, the 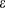 value is specified as the 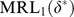 value (see the 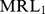 values in the second, fifth and eighth columns of Tables 2 and 3) of the optimal MRL-based EWMA 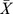 chart. Therefore, with the implementation of the optimization model (8)–(11) (see Section 4.1), the optimal DS 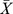 chart having the smallest 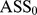 value, will also possess a reasonable 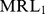 value which is similar to that of the optimal EWMA 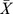 chart for the specified 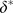. For the Shewhart 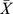 chart, it is designed to match the two MRL points of the EWMA 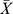 chart. Note that the two MRL points are the 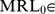
{250, 500} and a suitable 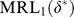 value, which is chosen such that the 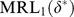 value of the Shewhart 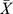 chart, with an appropriate 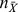, is as close as possible to that of the optimal EWMA 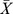 chart. For example, when 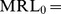
250, 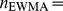
7 and 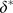 = 0.5, the optimal 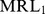 value for EWMA 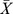 chart is five. Thus, both the DS 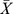 and Shewhart 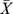 charts must have 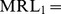
5 for 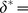
0.5.

From these tables, it is obvious that the optimal MRL-based DS 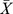 chart generally outperforms the optimal EWMA 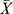 and Shewhart 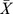 charts, in terms of the average sample size. Precisely, the 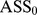 and 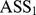 values of the optimal MRL-based DS 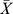 chart when 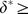
0.50 and 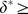
0.75, respectively, are lower than the corresponding values of 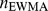 and 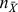. When compared with the optimal MRL-based EWMA 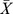 chart, the decrease in 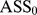 of the optimal DS 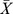 chart is around 36–85% when 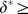
0.75; while the decrease in 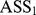 is around 13–82% when 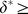
1.00. Tables 2 and 3 also reveal that there are substantial improvements in the 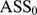 and 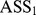 values of the optimal MRL-based DS 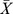 chart, in comparison to the 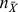 of the MRL-based Shewhart 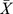 chart, where reductions of around 50–75% and 33–68%, respectively, exist, for 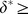
0.50. It is clear that from these two tables, the reduction of the out-of-control ASS is not as high as that of the in-control ASS. Also, by using the optimal MRL-based DS 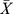 chart, we need a smaller sample size to detect moderate to large 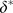 and a larger sample size to detect small 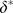. Generally, the optimal MRL-based DS 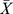 chart requires much smaller sample sizes on the average when the process is in-control and out-of-control and thus, using the chart reduces costs.

### 5.2 Study 2: The DS 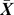 chart for minimizing the 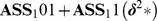


Table 4 summarizes the optimal (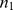, 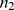, 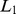, 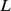, 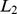) combination (listed in the first and second rows of each cell) of the DS 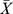 chart for minimizing the 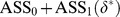, together with their respective (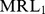, 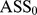, 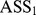) values (listed in the third row of each cell). The optimization model (12)–(15) in Section 4.2 is employed here. Therefore, it is ensured that all the optimal (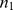, 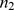, 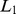, 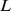, 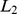) combinations in Table 4 attain 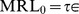
{250, 500} (constraint (13)) and 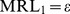 (constraint (14)) for a 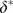. Here, the 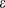 value is specified as the 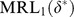 value of the optimal MRL-based EWMA 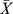 chart. In other words, both the optimal MRL-based DS 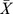 charts for minimizing the 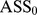 (see Study 1 of Section 5.1) and 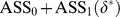 (see Study 2 of Section 5.2) have the same 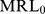 and 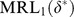 values.

**Table 4:** (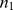, 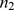, 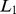, 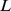, 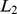) combination (first and second rows of each cell) and (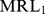, 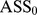, 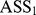) values (third row of each cell) of the DS 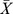 charts when 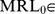
{250, 500} and 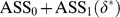 is minimized.

	MRL_0_ = 250			MRL_0_ = 500		
*δ* ^*^	*n*_EWMA_ = 3	*n*_EWMA_ = 5	*n*_EWMA_ = 7	*n*_EWMA_ = 3	*n*_EWMA_ = 5	*n*_EWMA_ = 7
0.50	(1, 19,	(1, 19,	(3, 17,	(1, 19,	(2, 18,	(3, 17,
	1.787, 5.561, 2.261)	1.473, 5.321, 2.477)	1.594, 4.955, 2.617)	1.767, 5.024, 2.558)	1.650, 5.147, 2.760)	1.506, 5.586, 2.915)
	(10, 2.405, 3.092)	(7, 3.675, 4.601)	(5, 4.885, 7.083)	(12, 2.466, 3.170)	(8, 3.781, 5.278)	(6, 5.246, 7.591)
0.75	(1, 9,	(1, 12,	(2, 11,	(1, 12,	(1, 15,	(2, 14,
	1.736, 5.334, 2.435)	1.538, 5.010, 2.503)	1.634, 4.400, 2.605)	1.764, 5.098, 2.640)	1.553, 5.455, 2.724)	1.650, 5.554, 2.811)
	(6, 1.742, 2.516)	(4, 2.488, 3.716)	(3, 3.125, 5.151)	(6, 1.934, 2.936)	(4, 2.806, 4.324)	(3, 3.384, 5.935)
1.00	(1, 5,	(1, 8,	(1, 8,	(1, 7,	(1, 8,	(2, 8,
	1.706, 5.029, 2.588)	1.283, 5.328, 2.692)	1.283, 5.328, 2.692)	1.770, 5.282, 2.750)	1.576, 4.997, 2.820)	1.731, 4.411, 2.899)
	(4, 1.440, 2.218)	(2, 2.596, 4.198)	(2, 2.596, 4.198)	(4, 1.537, 2.563)	(3, 1.920, 3.299)	(2, 2.668, 5.002)
1.25	(1, 3,	(1, 5,	(2, 5,	(1, 4,	(1, 6,	(2, 6,
	1.732, 4.216, 2.700)	1.611, 3.937, 2.646)	1.472, 4.272, 2.829)	1.760, 4.764, 2.885)	1.605, 4.281, 2.867)	1.452, 4.074, 3.054)
	(3, 1.249, 1.944)	(2, 1.535, 2.787)	(1, 2.705, 5.054)	(3, 1.314, 2.225)	(2, 1.650, 3.172)	(1, 2.878, 5.683)
1.50	(1, 3,	(1, 5,	(1, 5,	(1, 4,	(1, 5,	(1, 5,
	1.874, 3.541, 2.680)	1.351, 3.571, 2.789)	1.351, 3.571, 2.789)	1.907, 3.781, 2.855)	1.248, 4.535, 3.020)	1.248, 4.535, 3.020)
	(2, 1.182, 2.002)	(1, 1.882, 3.711)	(1, 1.882, 3.711)	(2, 1.226, 2.325)	(1, 2.060, 4.006)	(1, 2.060, 4.006)
1.75	(1, 2,	(1, 3,	(1, 3,	(1, 3,	(1, 4,	(1, 4,
	2.205, 4.297, 2.547)	1.578, 4.003, 2.766)	1.578, 4.003, 2.766)	2.201, 3.434, 2.926)	1.615, 3.605, 3.024)	1.615, 3.605, 3.024)
	(2, 1.055, 1.638)	(1, 1.343, 2.669)	(1, 1.343, 2.669)	(2, 1.081, 1.840)	(1, 1.424, 3.090)	(1, 1.424, 3.090)
2.00	(1, 3,	(1, 3,	(1, 3,	(1, 3,	(1, 3,	(1, 3,
	1.970, 4.016, 2.580)	1.970, 4.016, 2.580)	1.970, 4.016, 2.580)	1.890, 3.416, 3.097)	1.890, 3.416, 3.097)	1.890, 3.416, 3.097)
	(1, 1.146, 2.470)	(1, 1.146, 2.470)	(1, 1.146, 2.470)	(1, 1.174, 2.396)	(1, 1.174, 2.396)	(1, 1.174, 2.396)
2.50	(1, 2,	(1, 2,	(1, 2,	(1, 2,	(1, 2,	(1, 2,
	2.468, 3.021, 3.184)	2.468, 3.021, 3.184)	2.468, 3.021, 3.184)	2.451, 3.254, 3.281)	2.451, 3.254, 3.281)	2.451, 3.254, 3.281)
	(1, 1.022, 1.423)	(1, 1.022, 1.423)	(1, 1.022, 1.423)	(1, 1.026, 1.589)	(1, 1.026, 1.589)	(1, 1.026, 1.589)
3.00	(1, 2,	(1, 2,	(1, 2,	(1, 2,	(1, 2,	(1, 2,
	2.992, 3.437, 0.000)	2.992, 3.437, 0.000)	2.992, 3.437, 0.000)	3.000, 3.259, 2.578)	3.000, 3.259, 2.578)	3.000, 3.259, 2.578)
	(1, 1.004, 1.344)	(1, 1.004, 1.344)	(1, 1.004, 1.344)	(1, 1.003, 1.205)	(1, 1.003, 1.205)	(1, 1.003, 1.205)

Note that similar conclusions regarding the comparative performance of the in-control and out-of-control sample sizes among the three charts which are discussed for Tables 2 and 3, are obtained for Table 4. Thus, we compare the chart settings between the optimal MRL-based DS 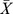 chart for minimizing the (i) 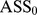 (see Tables 2 and 3 of Study 1) and (ii) 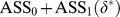 (see Table 4) in this Study 2. In Table 4, it is noticeable that some of the optimal (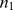, 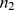, 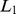, 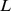, 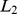) combinations are different from those shown in Tables 2 and 3. In addition, we found that the 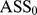 and 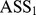 values in Studies 1 and 2 are fairly close to each other. We observe that there are some increments in the 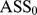 value and some decrements in the 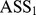 value for Study 2 as compared to that in Study 1. This is expected as we are minimizing both the 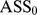 and 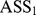 in Study 2. Note that the accuracies of all the results shown in Tables 1–4 have been verified with simulation.

## An Illustrative Example

In this section, we consider the example given by Carot et al. [30]. This example illustrates the implementation of the optimal MRL-based DS 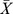 chart to monitor the amount of potassium sorbate to be added to a yoghurt manufacturing process. For the sake of comparison, the construction of the optimal MRL-based EWMA 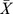 and Shewhart 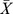 charts are also discussed in this section.

It is well known that potassium sorbate is a preservative, a bactericide and a fungicide. Hence, it is one of the basic ingredients to preserve a number of edible products. According to the public health institutions, the advisable amount of potassium sorbate to be added is 0.5–2.0 g per kg product. Thus, let 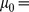
1.5 g and 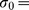
0.008 g as the desired process parameters of potassium sorbate in this yoghurt manufacturing process [29]. We initially generate the measurements of the first ten sampling times (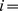
1 to 10) based on an in-control condition; whereas the measurements for the subsequent six sampling times (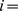
11 to 16) are generated with 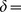
0.75. Table 5 tabulates various summary statistics for the DS 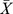, Shewhart 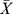 and EWMA 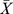 charts.

**Table 5:** Summary statistics of the simulated data for the amount of potassium sorbate (in grams, g) added to a yoghurt manufacturing process.

	DS *X*-bar chart				Shewhart *X*-bar chart	EWMA *X*-bar chart
Sample sizes	*n*_1_ = 1, *n*_2_ = 13				*n_X_*_-bar_ = 8	*n*_EWMA_ = 5
Sampling time, *i*	*X*-bar (1, *i*)	*Z*_1*i*_	*X*-bar (*i*)	*Z_i_*	*X*-bar_(Shewhart)_ (*i*)	*Z_i_*_(EWMA)_
1	1.5093	1.1567			1.5002	1.5009
2	1.5162	2.0279	1.5003	0.1494	1.5003	1.5021
3	1.4903	−1.2187			1.4997	1.4999
4	1.4957	−0.5422			1.4997	1.4985
5	1.5084	1.0502			1.5001	1.4987
6	1.5021	0.2635			1.5027	1.5002
7	1.4904	−1.1958			1.4997	1.4987
8	1.5028	0.3538			1.5019	1.5009
9	1.5044	0.5489			1.4959	1.5027
10	1.5013	0.1596			1.4965	1.4995
11	1.4982	−0.2227			1.5079	1.5050
12	1.4995	−0.0655			1.5008	1.5044
13	1.5125	1.5597			**1.5086**	1.5058
14	1.5199	2.4822	1.5061	**2.8433**	1.5074	1.5028
15	1.5080	1.0041			**1.5091**	**1.5068**
16	1.5144	1.8056	1.5075	**3.4898**	1.5044	1.5024

Remarks: The boldfaced values represent the out-of-control cases.

**Figure 2:**
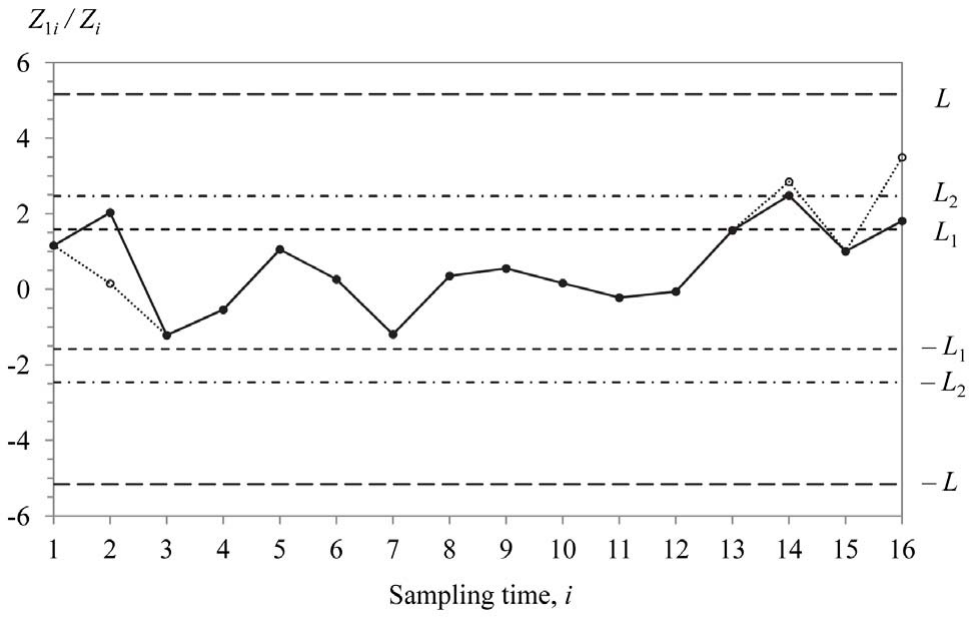
The DS 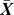 chart. The chart is used to monitor the amount of potassium sorbate to be added to a yoghurt manufacturing process. It produces the first out-of-control signal at sampling time 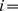
14.

**Figure 3:**
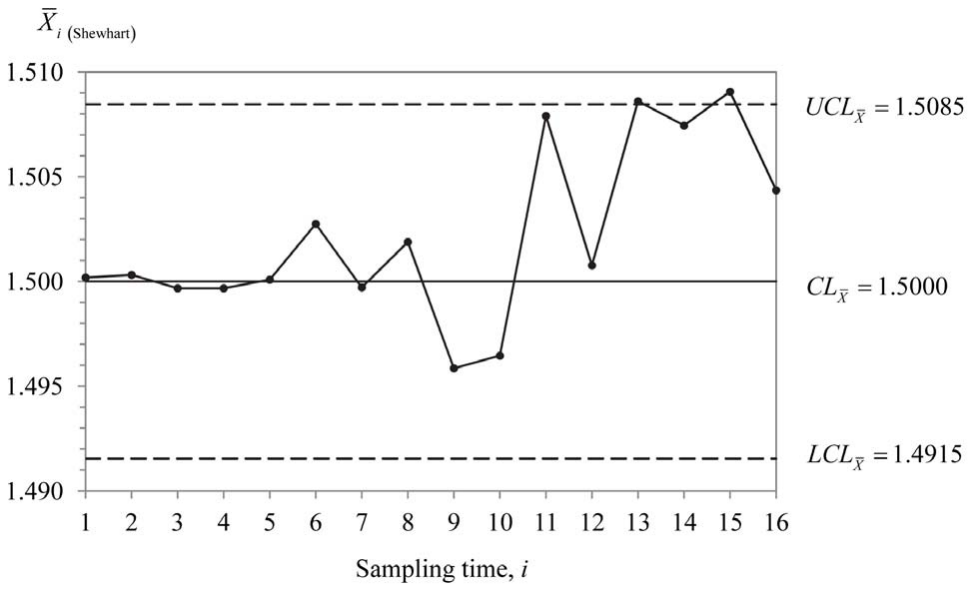
The Shewhart 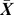 chart. The chart is used to monitor the amount of potassium sorbate to be added to a yoghurt manufacturing process. It produces the first out-of-control signal at sampling time 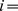
13.

**Figure 4:**
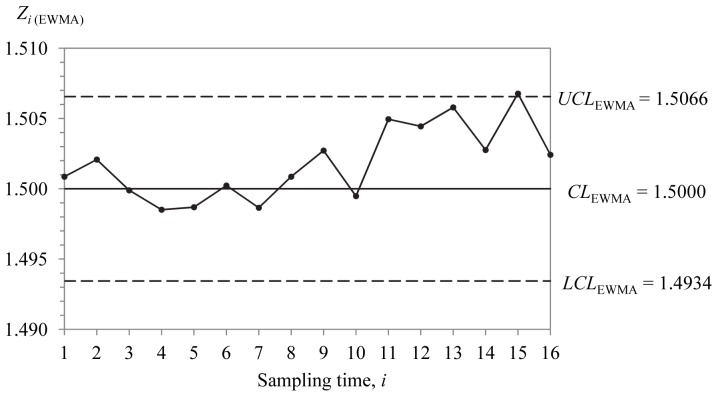
The EWMA 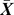 chart. The chart is used to monitor the amount of potassium sorbate to be added to a yoghurt manufacturing process. It produces the first out-of-control signal at sampling time 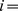
15.

Let us assume that 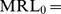
250 and 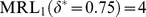 are desired. By referring to Table 2, the optimal chart parameters for the DS 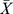, Shewhart 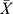 and EWMA 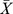 charts are (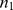, 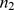, 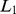, 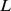, 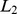) = (1, 13, 1.583, 5.163, 2.463), (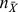, 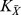) = (8, 2.992) and (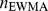, 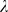, 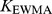) = (5, 0.550, 0.820), respectively. Figures 2 to 4 display the optimal MRL-based DS 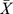, Shewhart 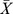 and EWMA 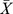 charts. The solid and hollow dots in Figure 2 represent 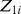 and 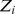 of the DS 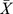 chart, respectively. Also, the values of the 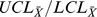 and 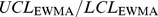 in Figures 3 and 4 are computed from Equations (16a) and (18a), respectively. Note that only the optimal chart parameters for the optimal MRL-based DS 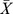 chart for minimizing the 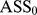 is considered in this example as both the optimal designs, i.e minimizing the 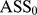 and 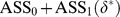, have almost similar 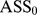 and 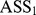 values.

From Figures 2 to 4, it is observed that the DS 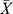, Shewhart 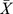 and EWMA 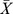 charts produce the first out-of-control signal at sampling time 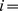
14 as 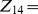
2.8433>
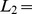
2.463, 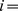
13 as 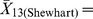
1.5086>
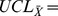
1.5085 and 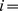
15 as 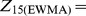
1.5068>
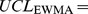
1.5066, respectively. This indicates that all the three charts have almost similar sensitivity in detecting 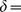
0.75. Concerning the number of observations sampled (from 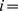
1 onwards; see Table 5), relatively less number of observations (40 observations) are required for the DS 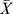 chart compared to the Shewhart 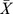 (104 observations) and EWMA 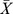 (75 observations) charts. It is apparent that the DS 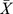 chart needs around 53% and 38% of the total sample size of the EWMA 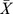 and Shewhart 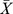 charts to detect the mean shift of 0.75.

## Conclusions

A good understanding of a control chart is vital as it helps to increase the quality engineers' confidence. Therefore, the MRL is chosen as the design measure in this paper because it is more readily comprehensible by the shop floor personnel and practitioners than the ARL. For completeness, this paper proposes two optimal designs of the MRL-based DS 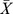 chart for minimizing the (i) 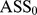 and (ii) 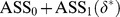, which are not yet available in the existing literature. Specific optimal chart parameters are provided in Tables 2 to 4 for these two optimal designs. These optimal chart parameters aid the practitioners to implement the optimal MRL-based DS 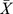 chart instantaneously.

From the comparative studies, it is found that the optimal MRL-based DS 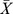 chart generally requires a smaller sample size on the average than the optimal EWMA 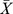 and Shewhart 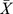 charts when the process is either in-control or out-of-control. The effectiveness of the optimal MRL-based DS 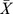 chart in reducing the sampling and inspection costs, provides a practical advantage for the practitioners in using this chart. Since both the optimal designs of the MRL-based DS 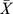 chart for minimizing the (i) 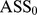 and (ii) 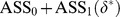, produce fairly close 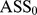 and 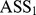 values, either one of these two optimal designs can be implemented in practice. The optimal MRL-based DS 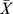 chart proposed in this paper provides an alternative to the SPC user and may stimulate more research interests in the area of the optimal MRL-based control charts.

## References

[1] Montgomery DC (2009) Statistical Quality Control: A Modern Introduction, 6^th^ ed. New York: John Wiley & Sons.

[2] Croasdale R (1974) Control charts for a double-sampling scheme based on average production run lengths. International Journal of Production Research 12: 585–592.

[3] Daudin JJ (1992) Double sampling *X̄* charts. Journal of Quality Technology 24: 78–87.

[4] Costa AFB, Machado MAG (2011) Variable parameter and double sampling *X̄* charts in the presence of correlation: The Markov chain approach. International Journal of Production Economics 130: 224–229.

[5] Khoo MBC, Lee HC, Wu Z, Chen CH, Castagliola P (2011) A synthetic double sampling control chart for the process mean. IIE Transactions 43: 23–38.

[6] Torng CC, Tseng CC, Lee PH (2010) Non-normality and combined double sampling and variable sampling interval *X̄* control charts. Journal of Applied Statistics 37: 955–967.

[7] He D, Grigoryan A (2002) Construction of double sampling *S*-control charts for agile manufacturing. Quality and Reliability Engineering International 18: 343–355.

[8] He D, Grigoryan A (2003) An improved double sampling *S* chart. International Journal of Production Research 41: 2663–2679.

[9] Lee PH, Chang YC, Torng CC (2012) A design of *S* control charts with a combined double sampling and variable sampling interval scheme. Communications in Statistics – Theory and Methods 41: 153–165.

[10] Lee PH, Torng CC, Wu JC, Tseng CC (2010) The effectiveness study of double sampling *S* charts application on destructive testing process. International Journal of Product Development 12: 324–335.

[11] He D, Grigoryan A (2006) Joint statistical design of double sampling *X̄* and *S* charts. European Journal of Operational Research 168: 122–142.

[12] Rodrigues AADA, Epprecht EK, Magalhães MSD (2011) Double-sampling control charts for attributes. Journal of Applied Statistics 38: 87–112.

[13] Torng CC, Lee PH, Liao NY (2009) An economic-statistical design of double sampling *X̄* control chart. International Journal of Production Economics 120: 495–500.

[14] He D, Grigoryan A, Sigh M (2002) Design of double- and triple-sampling *X̄* control charts using genetic algorithms. International Journal of Production Research 40: 1387–1404.

[15] Gupta BC, Walker HF (2007) Statistical Quality Control for the Six Sigma Green Belt. Milwaukee, Wisconsin: American Society for Quality, Quality Press.

[16] Torng CC, Lee PH, Liao HS, Liao NY (2009) An economic design of double sampling *X̄* charts for correlated data using genetic algorithms. Expert Systems with Applications 36: 12621–12626.

[17] Hsu LF (2004) Note on design of double- and triple-sampling *X̄* control charts using genetic algorithms. International Journal of Production Research 42: 1043–1047.

[18] Hsu LF (2007) Note on construction of double sampling *S*-control charts for agile manufacturing. Quality and Reliability Engineering International 23: 269–272.

[19] Das N (2009) A comparison study of three non-parametric control charts to detect shift in location parameters. International Journal of Advanced Manufacturing Technology 41: 799–807.

[20] Gan FF (1993) An optimal design of EWMA control charts based on median run length. Journal of Statistical Computation and Simulation 45: 169–184.

[21] Golosnoy V, Schmid W (2007) EWMA control charts for monitoring optimal portfolio weights. Sequential Analysis 26: 195–224.

[22] Thaga K (2003) Contributions to Statistical Process Control Tools. PhD Thesis. Winnipeg, Canada: University of Manitoba.

[23] Maravelakis PE, Panaretos J, Psarakis S (2005) An examination of the robustness to non normality of the EWMA control charts for the dispersion. Communications in Statistics – Simulation and Computation 34: 1069–1079.

[24] Palm AC (1990) Tables of run length percentiles for determining the sensitivity of Shewhart control charts for average with supplementary runs rules. Journal of Quality Technology 22: 289–298.

[25] Khoo MBC, Wong VH, Wu Z, Castagliola P (2012) Optimal design of the synthetic chart for the process mean based on median run length. IIE Transactions 44: 765–779.

[26] Low CK, Khoo MBC, Teoh WL, Wu Z (2012) The revised *m*-of-*k* runs rule based on median run length. Communications in Statistics – Simulation and Computation 41: 1463–1477.

[27] Nelder JA, Mead R (1965) A simplex method for function minimization. Computer Journal 7: 308–313.

[28] Zadeh LA (1963) Optimality and non-scalar-valued performance criteria. IEEE Transactions on Automatic Control 8: 59–60.

[29] Aparisi F, de Luna MA (2009) Synthetic *X̄* control charts optimized for in-control and out-of-control regions. Computers and Operations Research 36: 3204–3214.

[30] Carot V, Jabaloyes JM, Carot T (2002) Combined double sampling and variable sampling interval *X̄* chart. International Journal of Production Research 40: 2175–2186.

